# Supplementary material for: Volatile Dimethyl Disulfide from Guava Plants Regulate Developmental Performance of Asian Citrus Psyllid through Activation of Defense Responses in Neighboring Orange Plants
Source: Int J Mol Sci. 2022 Sep 7;23(18):10271. doi: 10.3390/ijms231810271 (PMC9499464; doi:10.3390/ijms231810271)
Supplement: Supplementary file 1 [file ijms-23-10271-s001.zip › ijms-1865907-supplementary.pdf]

Supplementary Materials

Supplementary Table S1 The release of volatiles from orange plants after exposure to dimethyl disulfide.

Volatile name [a]	RT (min)	RI	Exposure to sulfur volatiles (ng h^{-1} g^{-1} DW, n = 6)			
			Control	DMDS	10×DMDS	MeSA
α-Thujene [A, M]	6.71	928	Tr [b]	0.11 ± 0.04 b	0.12 ± 0.04 b	0.34 ± 0.04 a
α-Pinene [B, M]	6.86	936	Tr	0.14 ± 0.03 b	0.28 ± 0.12 b	2.24 ± 0.34 a
Sabinene [B, M]	7.64	976	17.28 ± 3.16 b	15.70 ± 2.65 b	19.85 ± 2.65 b	73.66 ± 11.70 a
β-Pinene [B, M]	7.71	979	Tr	0.15 ± 0.03 b	0.12 ± 0.03 b	2.36 ± 0.38 a
Myrcene [B, M]	7.95	992	2.05 ± 0.22 c	8.67 ± 0.61 b	8.96 ± 1.67 b	32.05 ± 12.21 a
Octanal [A]	8.18	1004	0.27 ± 0.04 b	0.57 ± 0.19 b	0.63 ± 0.15 b	8.68 ± 4.25 a
(*Z*)-3-Hexenyl acetate [B, GLV]	8.25	1007	0.24 ± 0.04 b	0.34 ± 0.06 b	0.51 ± 0.20 b	1.79 ± 0.33 a
Limonene [B, M]	8.71	1031	4.09 ± 0.64 b	8.80 ± 1.21 b	15.04 ± 6.00 b	40.13 ± 9.70 a
(*Z*)-β-Ocimene [B, M]	8.86	1038	0.25 ± 0.05 b	0.23 ± 0.03 b	0.42 ± 0.18 b	2.01 ± 0.11 a
(*E*)-β-Ocimene [B, M]	9.07	1049	2.27 ± 0.34 b	2.52 ± 0.64 b	4.13 ± 1.66 ab	12.29 ± 2.44 a
γ-Terpinene [A, M]	9.3	1049	Tr	0.15 ± 0.01 b	0.12 ± 0.02 b	1.24 ± 0.15 a
Sabinene hydrate [A, M]	9.49	1061	0.27 ± 0.03 b	0.22 ± 0.03 b	0.25 ± 0.04 b	1.40 ± 0.33 a
Linalool [B, M]	10.09	1102	0.34± 0.05 b	1.15 ± 0.21 b	3.20 ± 0.65 b	56.50 ± 12.90 a
Nonanal [A]	10.15	1105	0.94 ± 0.18 b	3.71 ± 1.10 b	10.13 ± 6.09 ab	23.07 ± 4.26 a
DMNT [B, Ht]	10.38	1117	0.74 ± 0.22 a	0.62 ± 0.19 a	0.40 ± 0.17 a	1.57 ± 0.30 a
Methyl salicylate [B]	11.92	1199	0.98 ± 0.19 c	5.53 ± 1.28 bc	13.20 ± 1.54 ab	16.60 ± 1.85 a
Decanal [A]	12.04	1207	0.81 ± 0.13 c	3.14 ± 0.58 bc	4.18 ± 0.52 b	17.50 ± 1.78 a
δ-Elemene [A, S]	14.44	1345	0.87 ± 0.19 b	0.98 ± 0.38 b	0.87 ± 0.43 b	3.37 ± 0.36 a
β-Bourbonene [A, S]	15.28	1396	0.32 ± 0.04 b	0.59 ± 0.39 b	0.79 ± 0.57 ab	2.16 ± 0.44 a
β-Elemene [B, S]	15.35	1401	5.00 ± 0.83 a	8.05 ± 1.50 a	5.49 ± 0.90 a	11.79 ± 1.78 a
α-Bergamotene [A, S]	15.71	1423	3.45 ± 0.51 a	3.37 ± 0.38 a	4.18 ± 0.77 a	6.77 ± 1.00 a
(*E*)-β-Caryophyllene [B, S]	15.86	1433	2.93 ± 0.26 b	5.77 ± 1.43 b	7.74 ± 1.85 b	16.80 ± 2.90 a
α-Humulene [B, S]	16.41	1467	0.34 ± 0.03 b	2.41 ± 0.45 ab	4.40 ± 1.02 ab	3.54 ± 0.49 a
Germacrene D [A, S]	16.83	1494	1.41 ± 0.31 b	3.64 ± 0.20 b	6.17 ± 1.59 b	14.44 ± 2.49 a
α-Farnesene [B, S]	17.12	1513	14.48 ± 1.80 a	12.89 ± 3.27 a	9.36 ± 2.35 a	12.95 ± 1.69 a
γ-Cadinene [A, S]	17.33	1527	8.21 ± 1.38 a	8.27 ± 0.90 a	7.79 ± 2.12 a	4.83 ± 1.42 a
TMTT [B, Ht]	18.17	1584	0.17 ± 0.05 a	0.15 ± 0.03 a	0.14 ± 0.02 a	0.30 ± 0.07 a
GLVs			0.24 ± 0.04 b	0.34 ± 0.06 b	0.51 ± 0.20 b	1.79 ± 0.33 a
Monoterpenes			22.04 ±3.78 c	37.86 ± 2.46 bc	52.49 ± 7.41 b	224.22 ± 32.75 a
Sesquiterpenes			37.00 ± 3.42 b	45.96 ± 3.66 b	46.80 ± 4.99 b	76.66 ± 6.09 a
Homoterpenes			0.91 ± 0.19 a	0.77 ± 0.18 a	0.54 ± 0.16 a	1.87 ± 0.31 a
Others (aldehyde/ester)			3.00 ± 0.35 c	12.95 ± 2.03 bc	28.14 ± 7.89 b	65.86 ± 7.03 a
Total			63.20 ± 6.88 c	97.88 ± 5.03 bc	128.48 ± 11.01 b	370.40 ± 41.40 a

[a] A = identification by comparison of mass spectrum and retention index (RI) according to NIST library (2016) and literatures. B = identification by comparison of authentic standard. GLV = C_6 green leaf volatiles, M = monoterpenes, S = sesquiterpenes, Ht = homoterpenes. [b] Tr = trace level. Data are shown as means ± SE (n = 6). Different letters in the same row indicate significant differences (Tukey's multiple comparisons, $p < 0.05$).

Supplementary Table S2 The number of semipolar differentially expressed metabolites (DEMs) of orange plants after exposure to dimethyl disulfide (related to Figure 7c).

Metabolite sets	DMDS vs. Control			10×DMDS vs. Control			MeSA vs. Control		
	Up	Down	Total	Up	Down	Total	Up	Down	Total
Benzene and derivatives	4	2	6	9	9	18	12	7	19
Carboxylic acids and derivatives	3	3	6	23	14	37	23	11	34
Cinnamic acids and derivatives	1	0	1	1	2	3	1	2	3
Coumarins and derivatives	1	0	1	3	1	4	3	1	4
Fatty Acyls	1	1	2	12	6	18	12	7	19
Flavonoids	2	1	3	4	6	10	4	4	8
Indoles and derivatives	2	1	3	3	2	5	3	3	6
Keto acids and derivatives	0	0	0	4	3	7	3	2	5
Organooxygen compounds	1	0	1	10	12	22	7	9	16
Organonitrogen compounds	3	1	4	3	1	4	5	0	5
Phenols	0	2	2	6	4	10	7	3	10
Prenol lipids	0	0	0	8	6	14	7	7	14
Steroids and derivatives	0	1	1	3	2	5	3	3	6
Others	3	6	9	18	31	49	17	21	38
Unclassified	10	3	13	48	29	77	41	31	72
Total number	31	21	52	155	128	283	148	111	259

Supplementary Table S3 Gene-specific primer pairs used for qRT-PCR.

Gene name	Forward primer (5'-3')	Reverse primer (5'-3')
PAL	CGTCGGTGCTAGTGGAGAG	TTCAGTGCGTGCTCAACAAG
SMT	TTCCGCAGTATACACCATCACC	ACAGCTCTCATGCAATTCGC
PR1 [a]	ACTGCAATCTTGTGCATTCG	TTCACCCACAGTTTCACAGC
FBOX [b]	TTGGAAACTCTTTCGCCACT	CAGCAACAAAATACCCGTCT
UPL7 [b]	CAAAGAAGTGCAGCGAGAGA	TCAGGAACAGCAAAAGCAAG

[a] PR1 primer reference from (Ibanez *et al.*, 2019). [b] FBOX and UPL7 are used as reference genes (Mafra *et al.*, 2012). Phenylalanine ammonia lyase (PAL), Salicylate-O-methyl transferase (SMT), Pathogenesis-related protein (PR1), Encoding a member of the F-box family (FBOX), Ubiquitin protein ligase 7 (UPL7).

Supplementary Table S4 UPHLC elution gradient and Q Exactive mass spectrometer settings. The gradient elution system consisted of (A) acetonitrile and (B) ammonium formate (5 mM in water) for ESI (−) mode, and (C) acetonitrile (0.1% formic acid) and (D) water (0.1% formic acid) for ESI (+) mode.

Setting	Gradient elution	ESI (−) mode		ESI (+) mode	
	procedure (min)	%A	%B	%C	%D
UHPLC	Initial	2	98	2	98
	1	2	98	2	98
	9	50	50	50	50
	12	98	2	98	2
	13.5	98	2	98	2
	14	2	98	2	98
	17	2	98	2	98
MS	Spray voltage			-2.50 kV (-) and 3.50 kV (+)	
	Sheath gas pressure			30 arb	
	Aux gas flow			10 arb	
	Capillary temperature			325°C	
	MS1 range			m/z 81-1000	
	MS1 resolving power			70000 FWHM	
	Number of data dependent scans per cycle			10	
	MS/MS resolving power			17500 FWHM	
	Normalized collision energy			30% (HCD model)	
	Dynamic exclusion time			Automatic	

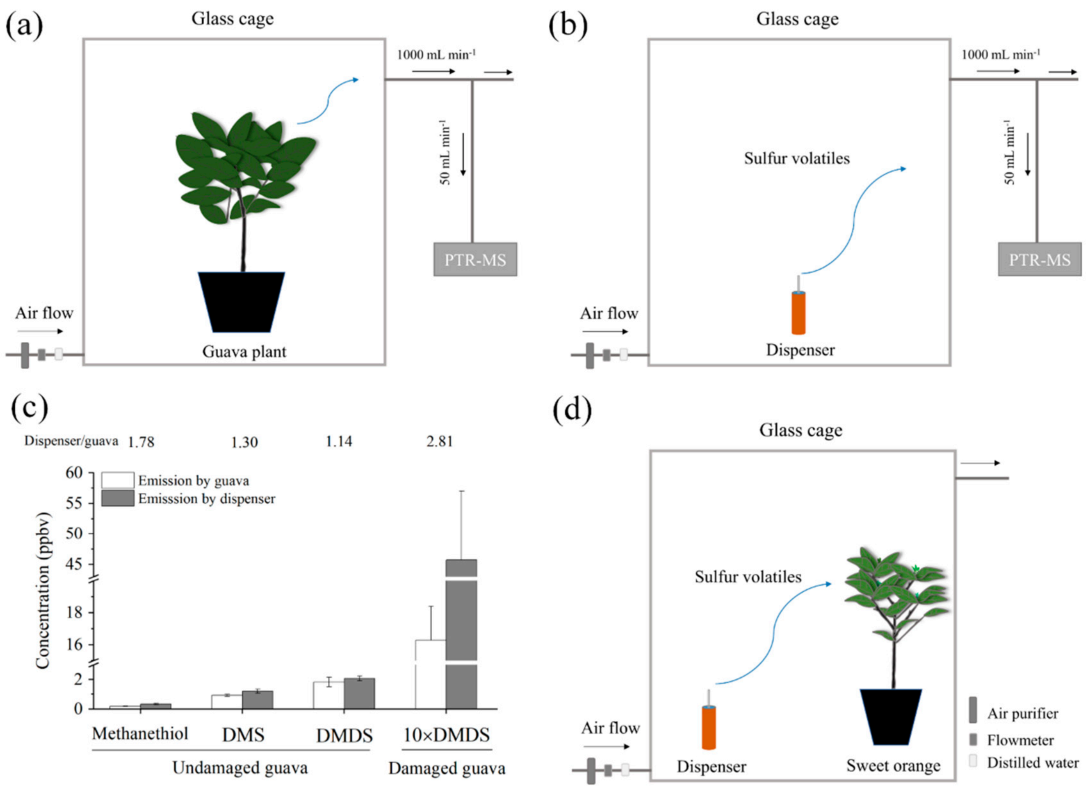

Supplementary Figure S1 Experimental setups for determination of guava sulfur volatiles and exposure of sweet orange plants to sulfur volatiles. (**a–b**) PTR-MS analysis of sulfur volatiles from (**a**) guava plants and (**b**) dispensers. (**c**) The ratios of sulfur volatiles released from dispensers and guava plants. In undamaged group, the concentrations of methanethiol, DMS and DMDS in the dispensers were 1, 5 and 10 μg μL^{-1}, thereby the concentrations in the glass cages were 0.34, 1.21 and 2.08 ppbv respectively at the second day. In mechanical damage group (10 × DMDS), the concentration of DMDS was 100 μg μL^{-1} in the dispenser and 45.71 ppbv in the glass cage, respectively. The concentrations of sulfur volatiles released by guava in the glass cages were shown in Figure 1. Thus, the emission of methanethiol, DMS, DMDS and 10 × DMDS from the dispensers were 1.78-, 1.30-, 1.14-, and 2.81-fold higher than that from guava plants. (**d**) A model of orange plant exposure to sulfur volatiles in the laboratory.
